# Access to Cancer Drugs in Canada

**DOI:** 10.3390/curroncol29100598

**Published:** 2022-10-12

**Authors:** Paul Wheatley-Price

**Affiliations:** Ottawa Hospital Research Institute, Department of Medicine, University of Ottawa, Ottawa, ON K1H 8L6, Canada; pwheatleyprice@toh.ca

Lung cancer, because of the multiple subtypes now identifiable and because of the myriad of new and effective therapies, provides fertile ground to highlight issues related to oncology drug access in Canada. Over the past few weeks in my clinical practice as a thoracic medical oncologist at an academic cancer centre in Canada, here are some examples of challenges I or my colleagues have faced with accessing cancer drugs.

I recently saw three new patients with MetEx14 skip mutation-positive non-small cell lung cancer (NSCLC). There are targeted drugs available for this. Tepotinib, approved by Health Canada and by the health technology assessment (HTA) process in Quebec (Institut national d’excellence en santé et en services sociaux (INESSS)), and indeed by the European Medicines Agency (EMA), the UK National Institute for Health and Care Excellence (NICE) and the US Food and Drug Administration (FDA), was nevertheless rejected by the CADTH (Canadian Agency for Drugs and Technologies in Health) HTA process [1]. I, therefore, could only prescribe tepotinib to one of the patients who had private insurance. The other two patients, without insurance, will receive capmatinib through a compassionate access program and with a Health Canada EPA application. The paperwork was filed just in time, as this access program is soon to close.

In contrast, a recent patient with positive Ret fusion lung cancer will be able to receive selpercatinib through a compassionate access program without resorting to a Health Canada EPA, as notice of compliance (NOC) has already been granted [2].

Furthermore, we recently had a deeply unpleasant experience with mesothelioma. The immunotherapy combination of nivolumab and ipilimumab is a new and effective therapy which was approved by HC and CADTH; however, a period of time was spent working through the pan-Canadian Pharmaceutical Alliance (pCPA), where price negotiations occurred [3]. While the company had an access program, and indeed extended that program, there was an uncomfortable period where the program had closed and funding was not yet secured, and yet, patients were referred to us with sarcomatoid mesothelioma, the particular subtype where the benefit is clearest from the new regimen. This resulted in multiple, repetitive and increasingly fraught emails to the Ministry of Health, urging rapid provincial coverage to be implemented, as in this case Ontario listed the treatment later than many other provinces.

If, in reading this Editorial, you also have had experiences of confusion, frustration, lack of understanding, or not knowing where to turn to access cancer drugs, then please read this Special Issue of *Current Oncology* titled ‘Access to Cancer Drugs in Canada’ (https://www.mdpi.com/journal/curroncol/special_issues/cancer_drugs_canada, accessed on 20 September 2022).

Perhaps start with the review article by Gotfrit et al. titled ‘The Pathway for New Cancer Drug Access in Canada’, which describes and defines the multiple steps to get a drug to market with public reimbursement [4]. This process begins with Health Canada, works through HTA assessments from CADTH or INESSS, then involves price negotiations with pCPA, before the drug is sent back to the provinces and territories for listing, with the additional role of the Patented Medicines Prices Review Board (PMPRB) thrown in for good measure.

The submitted articles to this Special Issue cover the spectrum of the process of drug approval.

Of course, when looking at time frames for accessing new drugs, the most common comparator nation is the USA, although their regulatory system is quite different. Nevertheless, Ho et al. looked at cancer drugs that received accelerated approval from the FDA in the US, and then reviewed what happened to those medications in the Canadian system, describing a median delay from FDA to Health Canada approval of over 9 months [5].

Dai and colleagues have published two papers in this issue from the CanREValue group (the Canadian Real-World Evidence for Value in Cancer Drugs). CanREValue was established in 2017 with stakeholders from across the spectrum of this field (patients, clinicians, payers, regulators) to establish the role of real-world evidence (RWE) to support cancer drug funding decisions. In the first of their papers, Dai et al. investigate drugs that are already funded, and with stakeholders they ran a simulation of how RWE might influence a reassessment of the funding of that drug. The exercise garnered new insights into the role of RWE, and how processes may be improved [6].

In their second paper, Dai et al. reported on the activities of the Reassessment and Uptake Working Group (RWG), one of the working groups that make up CanREValue [7]. Here, they describe strategies for using RWE to review funding decisions, which is an important component of an approval process when initial decisions are called into question, or need to be reassessed with new data.

A number of papers review challenges with our system. MacPhail and Snow highlight geographical differences [8]. The Canada Health Act entrusted Provinces and Territories to be responsible for healthcare for their populations. Thus, while many of the bodies described in the process are national (PMPRB, Health Canada, pCPA, etc.), the final funding decision remains with each province and the authors outline the disparities that subsequently emerge.

Gotfrit et al., in their second paper in the issue, highlight factors that are associated with positive provincial listings, concluding that a positive HTA recommendation is crucial, and that cancer type seems to be influential, However, the HTA recommendation does not seem to be influenced by the list price of the drug [9]. 

Sehdev However, the HTA recommendation does not seem to be influenced by the list price of the drug and Chambers also evaluated cancer drugs that went through the HTA process with the goal of identifying data points that went into an HTA decision, and how much certainty that would provide [10]. Out of 96 drug HTA submissions, they noted that in 57% of cases, a median overall survival (OS) for that drug was not available for the submission, and that caused a higher degree of uncertainty as reflected in the HTA decisions and explanations. As discussed in the CanREValue submissions, Sehdev and Chambers also discuss that the uncertainty in some HTA decisions highlights the need for an effective resubmission process. Jenei et al. also discuss similar themes in their paper ‘Describing Sources of Uncertainty in Cancer Drug Formulary Priority Setting across Canada’ after interviewing senior officials in multiple provinces [11]. In particular, they highlight the challenges brought about by the myriad of new cancer drugs, with early data or in small populations, and suggest some opportunities including the use of RWE and the consideration of risk agreements.

Changes are underway in the Canadian system, and some of these naturally raise questions. Binder et al., in their paper ‘Health Technology Assessment Process for Oncology Drugs: Impact of CADTH Changes on Public Payer Reimbursement Recommendations’, specifically highlight an issue of QALY (quality-adjusted life years), which is a health economics tool whereby a ‘cost per QALY’ can be estimated to provide guidance on whether a new drug is affordable and whether it should be reimbursed [12]. In their paper, they describe a change in 2020 whereby the ‘cost per QALY’ threshold was reduced, and confirm that this has been observed in HTA submissions since then. This then feeds into the pCPA cost negotiation, and while it would be lovely to have all our cancer drugs at filthy-cheap prices, the reality is that if drug prices are suppressed too much then companies may withdraw their products from the market entirely. Indeed we have now observed a number of cases where pCPA agreements have not been reached, or taken far too long. On a similar theme, Kaplan et al. reflect on the new federal pricing policies brought in by the Canadian federal government through the PMPRB process, and expound on the potential unanticipated consequences of these changes [13]. In particular, they express concern as to whether forced lower prices will reduce clinical trials in Canada, and ultimately reduce access to cancer drugs.

Related to this, Glennie et al., in their paper ‘Closing the Gaps to Timely Patient Access: Perspectives on Conditional Funding Models’ propose and discuss models whereby access to medications can be secured for patients while the regulatory process works through its often timely mechanism [14]. They provide examples from other jurisdictions, such as the UK and France, that are being established for the purpose of enabling patients to receive effective therapies without jeopardizing the robustness of the regulatory system.

With all of these papers discussing the process of cancer approval, of course there are drugs that are not approved. In their paper ‘Current Attitudes toward Unfunded Cancer Therapies among Canadian Medical Oncologists’, Wong et al. surveyed clinicians about how they address these clinical situations, which as I outlined at the beginning of this Editorial, are frequent [15].

In summary, this Special Issue ‘Access to Cancer Drugs in Canada’ is a timely overview of a complex and evolving system. Countries throughout the world are grappling with how to approve and fund an increasing number of effective but expensive cancer drugs, and in this respect Canada is no different. In many aspects, Canadians can be fortunate in the high level of cancer care they receive, but as the authors of this collection of manuscripts attest, there is work to do.
